# Pediatric Clostridium septicum infections: a systematic review and case-based perspective

**DOI:** 10.1186/s12887-026-07544-5

**Published:** 2026-08-22

**Authors:** Frederike Bieling, Jasmin Özcan, Julius Sommer, Patrick Morhart, Steven Hébert, Manuel Besendörfer, Sonja Diez

**Affiliations:** 1 Friedrich-Alexander-Universität (FAU) Erlangen-Nürnberg, Department of Pediatric Surgery, University Hospital Erlangen, Erlangen, Germany; 2 Friedrich-Alexander-Universität (FAU) Erlangen-Nürnberg, Department of Pediatrics, University Hospital Erlangen, Erlangen, Germany; 3 Friedrich-Alexander-Universität (FAU) Erlangen-Nürnberg, Microbiology Institute – Clinical Microbiology, Immunology and Hygiene, University Hospital Erlangen, Erlangen, Germany; 4 Friedrich-Alexander-Universität (FAU) Erlangen-Nürnberg, Bayreuth Hospital, Institute for University Teaching and Research Medical Campus Upper Franconia MCO, Department of Children and Adolescents, Bayreuth Hospital, Bayreuth, Germany

**Keywords:** Infectious disease, Clostridium septicum, Clostridial myonecrosis, Intussusception, STEC-HUS, Surgical debridement, Sepsis, Systematic review

## Abstract

**Context:**

Pediatric Clostridium septicum infections are extremely rare but often fulminant and rapidly progressive. They may manifest as clostridial myonecrosis, sepsis, or with gastrointestinal involvement. Systematic data on presentation, management, and outcomes remain scarce.

**Objective:**

To systematically review published pediatric cases of C. septicum infection, focusing on clinical presentation, underlying conditions, management, and outcome as an update to the review by Smith-Slatas et al. (2006). In particular, to assess how frequently gastrointestinal involvement occurs in this disease and whether its presence is associated with poorer outcomes. Furthermore, to highlight the diagnostic challenges and therapeutic urgency of this condition through a fatal case.

**Data sources:**

PubMed, Scopus, Web of Science, and the Cochrane Library were searched for reports from May 2006 to May 2025.

**Study selection:**

Studies describing patients under 18 years with confirmed C. septicum infection were included.

**Data extraction:**

Variables included infection site, underlying disease, treatment, and outcome. A previously unpublished fatal case of pediatric clostridial myonecrosis with ileocolic intussusception was added. Fisher’s exact test was used for statistical analysis.

**Results:**

Only 18 pediatric cases published since 2006 were found; 44% had clostridial myonecrosis and 50% gastrointestinal involvement. STEC-HUS occurred in 50%. Compared with a 2006 review, rates of STEC-HUS, surgical intervention, overall survival, and absence of gastrointestinal involvement have increased. Surgical debridement was strongly associated with survival (*p* < 0.01), whereas gastrointestinal involvement was associated with fatal outcome (*p* = 0.014). The reported child deteriorated rapidly after initial intussusception diagnosis and died despite maximal intensive care.

**Limitations:**

Very few cases are available because the disease is exceptionally rare; the analysis is retrospective.

**Conclusions:**

Surgical source control is associated with improved survival. Gastrointestinal involvement is a major risk factor for poor outcome and may include intussusception.

**Supplementary Information:**

The online version contains supplementary material available at 10.1186/s12887-026-07544-5.

## Background

Clostridial myonecrosis (CM), commonly known as gas gangrene, is a rare but rapidly progressive and often fatal soft tissue infection caused by toxin-producing, anaerobic, spore-forming gram-positive bacilli, most notably *Clostridium (C.) perfringens*, *C. septicum*, and *C. histolyticum* [1]. These pathogens can cause extensive tissue necrosis and systemic toxicity within hours of symptom onset, requiring urgent recognition and immediate intervention. Although CM is primarily known from adult surgical and trauma contexts, its occurrence in children is exceedingly rare [2–16]. The highest mortality is observed when *Clostridium septicum*, which was also present in our case, is the causative bacterium [15]. *Clostridium* spp. are widely found in soil, water, and the human gastrointestinal tract, and are capable of inducing fulminant necrosis and systemic toxicity via exotoxin release [17–19]. *C. septicum* can be detected in the feces of 2.8% of healthy individuals [15, 20]. As an obligate anaerobe, *Clostridium* species thrives in hypoxic environments, making tissue hypoperfusion or ischemia a central risk factor for infection [15, 21].

Despite advances in critical care and antimicrobial therapy, mortality remains high even with aggressive surgical and medical management [15, 17, 22]. Early diagnosis is essential, yet clinical presentation may be nonspecific in the early stages. Pediatric cases often evolve rapidly, with fulminant soft tissue involvement, systemic inflammatory response, and multiorgan failure within a narrow therapeutic window.

A larger case series published by Smith-Slatas et al. in 2006 reviewed 47 pediatric cases of *C. septicum* infection, of which 22 (47%) were associated with myonecrosis. The authors identified three major clinical contexts: neutrophil dysfunction (e.g., leukemia or chemotherapy), bowel ischemia, and trauma [15]. The mortality rate among children with myonecrosis was 57%, and only 35% of those with gastrointestinal involvement survived. Approximately 10% of the reported cases were spontaneous (i.e., nontraumatic and not hospital-acquired), which are especially lethal due to their insidious onset and rapid deterioration [15]. Smith-Slatas et al. also noted that gastrointestinal symptoms—vomiting, diarrhea, hematochezia, anorexia, and acute abdominal pain—were frequent but nonspecific early signs [2]. Radiologically, gas in the myofascial plane is a hallmark finding, although it may not be present in the early stages [1, 3–16].

Since the publication of the seminal review by Smith-Slatas et al. in 2006, the literature on pediatric C. septicum infection has remained limited to isolated case reports, without any updated systematic evaluation of reported cases. Their review comprehensively characterized the clinical spectrum of pediatric disease up to 2006 and has since served as the principal reference in this field. Building on this important foundation, the present review aims to provide an updated systematic analysis of subsequently published pediatric cases.

This review summarizes the current literature on *C. septicum* infections in children, with specific attention to cases of CM. The goal is to identify clinical patterns, elucidate risk factors, and define factors associated with survival. A particular focus was placed on assessing how frequently gastrointestinal involvement occurs and whether its presence is associated with poorer outcomes. A fatal case of pediatric CM is presented as an illustrative example of the disease’s fulminant progression and to underscore the narrow therapeutic window in affected children.

## Main text

### Methods

A systematic literature review was conducted to identify pediatric cases of *Clostridium septicum* infection published between May 2006 and May 2025. The review was conducted in accordance with the PRISMA guidelines. The completed PRISMA checklist is provided as supplementary material. As the review by Smith-Slatas et al. comprehensively summarized pediatric cases published up to 2006, the present literature search was intentionally restricted to studies published from 2006 onward to provide an updated analysis of subsequently reported cases. This approach was chosen to build upon the existing systematic work while specifically evaluating more recent clinical patterns, management strategies, and outcomes. The literature search strategy was predefined and performed systematically in PubMed, Scopus, Web of Science, the Cochrane Library, and ClinicalTrials.gov. In all databases the following Boolean search string was used: ((“Clostridium septicum”[Title/Abstract]) OR (“clostridial myonecrosis”[Title/Abstract])) AND ((child[Title/Abstract]) OR (pediatric[Title/Abstract]) OR (infant[Title/Abstract]) OR (adolescent[Title/Abstract])). Equivalent search terms adapted to the respective database syntax were applied in Scopus, Web of Science, the Cochrane Library, and ClinicalTrials.gov. Searches were restricted to English-language publications involving human pediatric patients and published between January 2006 and May 2025 [2–14, 16]. The reference lists of all included publications were manually screened for additional eligible studies. However, no further relevant pediatric cases were identified beyond those retrieved through the predefined database search strategy.

Inclusion criteria comprised the following: patients under 18 years of age; confirmed *Clostridium septicum* infection; availability of clinical details regarding presentation, treatment, and outcome. Article type was not restricted; however, only case reports, case series, and one literature review based on case reports were identified. Exclusion criteria included adult patients, infections caused by other *Clostridium* species, review articles without original case data, and reports lacking differentiation between species or relevant clinical information.

One author screened all titles and abstracts, followed by full-text assessment of potentially eligible studies; the complete eligibility assessment and data extraction were subsequently reviewed by a second independent expert. Data were extracted using a standardized template and included age, sex, initial signs and symptoms, anatomical site of infection, underlying disease, suspected pathophysiological trigger, antimicrobial therapy, time to antibiotic administration, surgical management, time to debridement, and outcome (see Table 1).

**Table 1:** Overview of 18 pediatric cases of Clostridium septicum infection published since 2006. Clinical features, infection localization, suspected pathophysiologic triggers, therapeutic timelines, and outcomes are summarized. Cases were extracted from peer-reviewed publications. Clostridial myonecrosis (CM) was defined by clinical, imaging, or intraoperative findings of gas-forming muscle necrosis

Authors	Journal	Age	Sex	Initial signs and symptoms	Infected localisation	Underlying disease	Trigger suspected by authors	Antibiotic use	CM	Time to antibiotic	Surgical debridement	Time to surgical debridement	Outcome
Bieling et al. 2026	X	7	F	Right lower abdominal pain	Bowl, left flank and hemiabdomen, both upper thighs, retroperitoneum	STEC-HUS	Mucosal injury due to HUS	Meropenem, Vancomycin, Cefotaxime, Penicillin G, Clindamycin	Yes	< 24 h	Yes	> 24	Lethal
Chang et al. 2007	Ann Trop Paediatr	5	M	Right lower abdominal pain	Ileum and colon	None	Not specified	Not specified	No	Not specified	Yes	< 24 h	Lethal
Hunley et al. 2008 [9]	Pediatr Nephrol	1	M	Diarrhea with blood, decreased intake, edema	Right arm, volar compartment, shoulder region	STEC-HUS	Mucosal injury due to HUS and catheter-related seeding	Cefazolin, Gentamicin, Metronidazol, Cefepime, Vancomycin, Clindamycin, Penicillin	Yes	<24 h	Yes	<24 h	Not lethal
Wiersema et al. 2008 [23]	Orthopedics	16	F	Right arm discomfort and swelling	Right arm, right thigh, left thigh	None reported	Spontaneous	Broad-spectrum antibiotics (not specified)	No	<24 h	Yes	<24 h	Not lethal
Martin et al. 2012 [12]	Brain Pathology	2	M	Bloody diarrhea	Brain (pneumocephalus), colon, spleen, retroperitoneum	STEC-HUS	Mucosal injury due to HUS	Not specified	No	Not specified	No	None performed	Lethal
Pinzon-Guzman et al. 2013 [5]	Journal of Pediatric Surgery	8	F	Bilateral leg swelling and soreness, crepitus	Both lower extremeties, Ischemic ascending colitis	*C. difficile* Enterocolitits (post AB) after Streptococcal pharyngitis	Mucosal injury due to C. difficile colitis	Penicillin, Clindamycin	Yes	<24 h	Yes	<24 h	Not lethal
Sadarangani et al. 2014 [14]	Pediatr Infect Dis J	0	M	Apnea, bradycardia, feeding intolerance	Brain (frontal lobes, ventricles)	NEC, Prematurity	Mucosal injury due to NEC	Vancomycin, Meropenem, Metronidazol	No	<24 h	Yes	< 24 h	Not lethal
Kiser et al. 2014 [11]	J Pediatr Orthop	9	F	Abdominal pain and diarrhea	Left thigh, colon (leocecal valve to mid-transverse colon)	*C. difficile* Enterocolitits (post AB) after Streptococcal pharyngitis	Mucosal injury due to *C. difficile* colitis	Piperacillin-Tazobactam, Vancomycin, Metronidazole, Clindamycin, Penicillin, Meropenem	Yes	<24 h	Yes	< 24 h	Not lethal
Iddings et al. 2017 [10]	Clinical Nephrology	5	M	Abdominal pain andnon-bloody diarrhea	Colon (perforation), peritoneum	STEC-HUS, New-onset type 1 diabetes mellitus	Mucosal injury due to HUS	Metronidazol, Clindamycin, Ampicillin, Piperacillin-Tazobactam, Fluconazole	No	<24 h	Yes	<24 h	Not lethal
Engen et al. 2017 [8]	Pediatrics	3	F	Bloody diarrhea, fever, abdominal tenderness	Brain parenchyma, hemorrhagic necrosis of colon, abdominal wall	STEC-HUS	Mucosal injury due to HUS	Broad-spectrum antibiotics (not specified)	Yes	< 24 h	No	None performed	Lethal
Engen et al. 2017 [8]	Pediatrics	4	F	Abdominal pain, fever	Bloodstream (bacteremia)	STEC-HUS	Mucosal injury due to HUS	Metronidazole, Meropenem, Clindamycin	No	<24 h	No	None performed	Not lethal
Engen et al. 2017 [8]	Pediatrics	6	F	Abdominal pain, fever	Bloodstream (bacteremia)	STEC-HUS	Mucosal injury due to HUS	Vancomycin, Gentamicin, Penicillin	No	<24 h	No	None performed	Not lethal
Parmar et al. 2021 [13]	JAMMI	14	F	Abdominal pain, fever, vaginal bleeding	Right arm (volar compartment)	Burkitt’s lymphoma (chemo)	Alternated mucosal barrier due to neutropenia	Ceftazidime, Metronidazole, Penicillin G	Yes	<24 h	Yes	<24 h	Not lethal
Cirillo et al. 2023	Journal of Nephrology	5	F	Bloody diarrhea, general malaise	Brain (frontal abscess)	STEC-HUS	Mucosal injury due to HUS	Meropenem, Vancomycin, Amikacin, Metronidazol	No	<24 h	Yes	No	Lethal
Anzinger et al. 2023 [6]	J Surg Case Rep	8	F	Abdominal pain, fever coffee-ground emesis	Terminal ileum, caecum, ascending colon, right leg	Cyclic neutropenia	Alternated mucosal barrier due to neutropenia	Ceftriaxone, Vancomycin, Piperacillin-Tazobactam, Vancomycin, Clindamycin	Yes	<24 h	Yes	<24 h	Not lethal
Herrin et al., 2015 [24]	J Pediatr Surg Case Rep	17	M	Acute abdominal pain, fever, emesis, diffuse peritonitis	Ileum, bloodstream	Relapsed testicular embryonal rhabdomyosarcoma (on chemo including temsirolimus)	Mucosal breakdown and bowel ischemia under mTOR inhibition (temsirolimus)	Not specified	Yes	Not specified	Yes	< 24 h	Not lethal
Grover et al. 2019 [25]	Anaerobe	0	F	Fever, swelling of left knee	Left knee joint	None specified	Possibly direct inoculation from minor trauma	Ceftriaxone, Cloxacillin, Metronidazole	No	<24 h	Yes	> 7 days	Not lethal
Lim et al. 2020 [26]	J Infect Dis Epidemiol	2	M	Bloody diarrhea, lethargy, pallor, oliguria	Bloodstream (bacteremia)	STEC-HUS	Mucosal injury due to HUS	Ceftriaxone, Metronidazole	Yes	<24 h	No	None performed	Not lethal

*Abbreviations: CM* Clostridial myonecrosis, *NEC* Necrotizing enterocolitis, *STEC-HUS* Hemolytic uremic syndrome associated with Shiga toxin-producing *Escherichia coli,* *AB* Antibiotics, *AKI* Acute kidney injury, *None performed* Patient received no surgical source control

“Time to antibiotic treatment” and “Time to surgical debridement” indicate intervals between onset of CM-typical symptoms (e.g., crepitus, skin changes, systemic deterioration) and respective interventions. “Yes” under “Surgical therapy” refers to surgical exploration with or without debridement. “Outcome” reflects reported survival status at hospital discharge

Definitions of clostridial myonecrosis were based on clinical, imaging, or intraoperative findings of gas-forming tissue necrosis. The case reported in this review was included using the same criteria for comparative purposes. No formal quality assessment tool was applied, given the descriptive nature and limited number of eligible reports.

The inclusion of case reports inherently results in methodological and clinical heterogeneity. Primary data were extracted directly from all eligible published articles without reinterpretation of secondary analyses, thereby minimizing the risk of bias introduced by the review process itself. All identified case reports meeting the predefined eligibility criteria were included without selective exclusion, reducing selection bias and ensuring comprehensive data capture. The dataset is considered robust because all available case reports were included in the analysis.

Data were recorded as quantitative variables or categorial factors and analyzed according to subgroups. Statistical analysis was conducted using IBM SPSS Statistics (Version 29.0.2.0 (171)). In order to compare subgroups according to survival or publication, chi-square tests or Fisher’s exact test have been performed, as appropriate. In general, a test result with a p-value less than or equal to 0.05 was considered statistically significant.

## Results

### Literature review

According to the searching criteria, 15 publications could be identified, 14 case reports, 1 case series and 0 reviews, compiling for 17 cases since 2006.

### Review of most recent pediatric *C. septicum* infections

Since 2006, 18 pediatric *C. septicum* infections have been published (summarized in Table 1 and including one prior unpublished patient of our hospital, see below), including both, cases with and without myonecrosis [3–6, 6, 7, 7–16, 24–26]. Patients presented with a median age of 5 years and sex ratio was distributed toward female patients (f: m *n* = 11:7). Among all 18 cases, 44% (8/18) presented with CM, 50% (9/18) had gastrointestinal involvement, and 50% (9/18) were associated with hemolytic uremic syndrome (HUS), most often in the context of Shiga toxin-producing *E. coli* (STEC) infection. In those 18 recent cases none of the clinical variables could be identified in association with outcome of patients.

### Comparison to 2006 review of Smith-Slatas et al.

To contextualize our findings, we compared the recent 18 pediatric *C. septicum* cases included in this review with the 47 cases summarized by Smith-Slatas et al. in their landmark 2006 publication. In the earlier cohort, gastrointestinal involvement was reported in 85% of cases, whereas in our post-2006 series this applied to 50% (*p* = 0.003). Notably, STEC-HUS was identified in 50% of our cases, compared to only 10% in the Smith-Slatas group (*p* < 0.01). Surgical debridement was performed more frequently in the recent cases (72% vs. 49%, *p* = 0.047), and the overall lethality was markedly lower (27% vs. 57%, *p* = 0.032). Results are summarized in Table 2.

**Table 2:** Comparison of pediatric Clostridium septicum cases in children reported by Smith-Slatas et al. (before 2006 [15]), and Bieling et al. (2006–2025) regarding gastrointestinal involvement, association with STEC-HUS, rate of surgical therapy, and survival

	Bieling et al. (*n* = 18)	Smith-Slatas et al. (*n* = 47)	*p*-value
GI involvement of infection [*n* (%)]			
yes	9 (50%)	40 (85%)	0.003
no	9 (50%)	7 (15%)	
STEC-HUS [n (%)]			
yes	9 (50%)	5 (11%)	< 0.001
no	9 (50%)	42 (89%)	
Surgical therapy [n (%)]			
yes	13 (72%)	21 (45%)	0.047
no	5 (28%)	26 (55%)	
Survival [n (%)]			
yes	13 (72%)	20 (43%)	0.032
no	5 (28%)	27 (57%)	

*Abbreviations: GI* Gastrointestinal, *STEC-HUS* Hemolytic uremic syndrome associated with Shiga toxin-producing Escherichia coli

To further examine prognostic factors, we performed a statistical analysis combining all 65 pediatric cases from our cohort and the previously published series by Smith-Slatas et al. [15]. Two variables were significantly associated with survival. First, the presence of gastrointestinal involvement was negatively associated with outcome: while 30 of 32 patients (94%) with fatal courses had gastrointestinal involvement, only 19 of 33 survivors did (58%, *p* < 0.01). Second, surgical debridement was strongly associated with survival. Among patients who underwent surgery, 26 survived and only 8 died, whereas among those not surgically treated, 24 died and only 7 survived (*p* < 0.01). In contrast, no significant association was found between outcome and STEC-HUS status, although a trend could be noted (*p* = 0.504).

### Case presentation

**Fig. 1:**
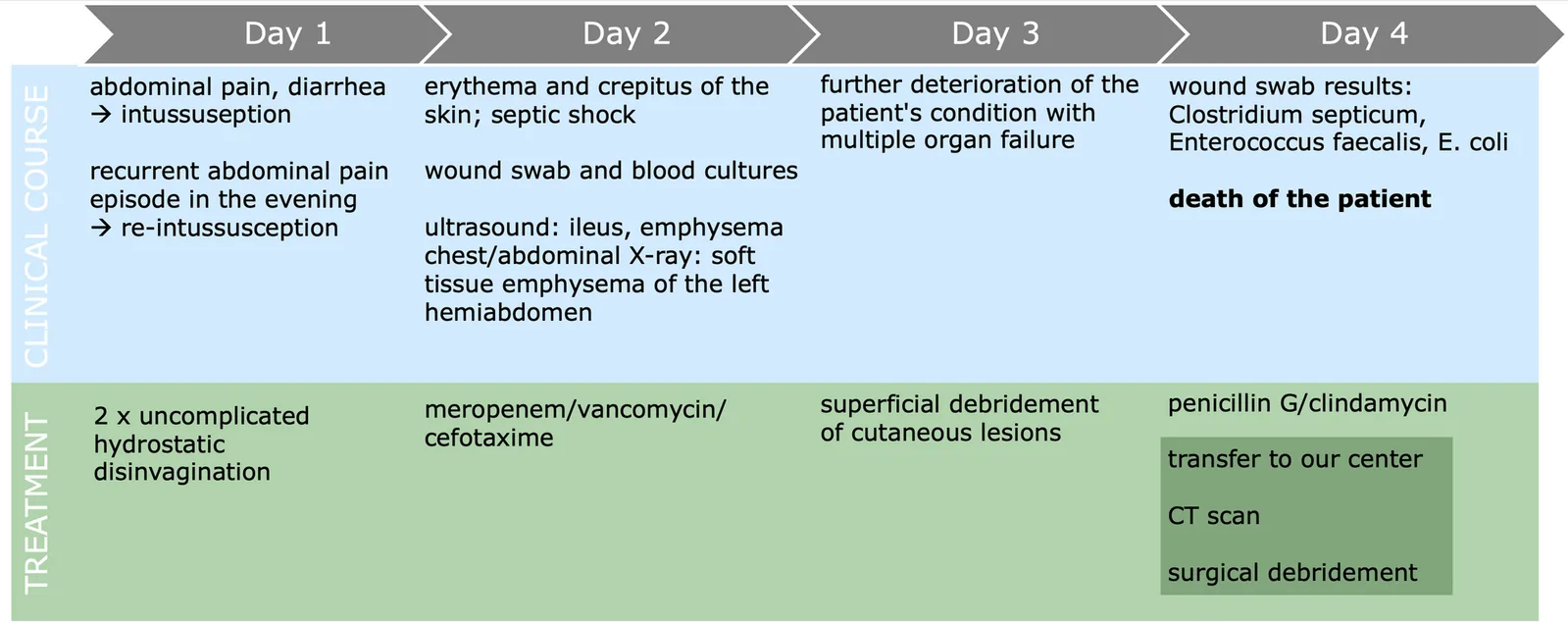
Clinical timeline of the presented case, illustrating the progression from ileocolic intussusception to fulminant Clostridium septicum sepsis, surgical intervention, and death

The case report was conducted in accordance with the CARE guidelines and approved by the local ethics committee. Written informed consent for publication was obtained from the child’s legal guardians after comprehensive explanation of the nature and purpose of the publication. The clinical course is summarized in Fig. 1.

### Clinical course prior to referral

The patient, a previously healthy seven-year-old girl initially presented with abdominal pain, followed by fever and diarrhea. Six days after symptom onset, ultrasound revealed an ileocolic intussusception, and she was referred to a pediatric surgical center. On admission, ultrasound confirmed a 3.6 cm ileocolic intussusception. Hydrostatic disinvagination was successfully performed twice due to recurrent re-invagination later the same day.

The following morning, progressive cutaneous erythema and palpable subcutaneous crepitus were noted on the back and flank. Concurrently, the child’s clinical status deteriorated rapidly, prompting transfer to the pediatric intensive care unit with a working diagnosis of sepsis. Imaging studies revealed progressive subcutaneous emphysema involving the left abdominal wall and thorax, without evidence of free intraabdominal air or pneumatosis intestinalis. These findings were based on ultrasound and plain radiography. A suspected diagnosis of a necrotizing soft tissue infection with gas formation was made.

In the intensive care setting, the patient developed severe hemodynamic instability, acute renal failure, respiratory failure requiring intubation, and progressive skin hemorrhages. Broad-spectrum antibiotic therapy was initiated (meropenem, vancomycin and cefotaxime). Supportive care included catecholamine infusions, corticosteroids, immunomodulation with anakinra, and granulocyte colony-stimulating factor.

Microbiological testing of skin lesions confirmed the presence of *Clostridium septicum*, *Escherichia coli* and *Enterococcus faecalis* (culture results on day 4 of admission). Following identification of *C. septicum*, the antibiotic regimen was expanded to include high-dose penicillin G and clindamycin. Because of progressive multi-organ failure and refractory respiratory insufficiency, the patient was transferred to our institution on day 4 of admission.

Laboratory findings confirmed severe inflammatory response. Disseminated intravascular coagulation developed on day 2 of admission. Liver enzymes increased dramatically (GOT/AST up to 14,786 U/L, GPT/ALT up to 3,235 U/L), and creatine kinase peaked at 45,527 U/L, consistent with extensive muscle destruction. Concurrently, LDH levels exceeded 6,500 U/L and Pro-BNP surpassed 35,000 pg/mL, underscoring severe systemic inflammation, tissue breakdown, and multiorgan involvement.

### Clinical course following transfer to our institution

**Fig. 2:**
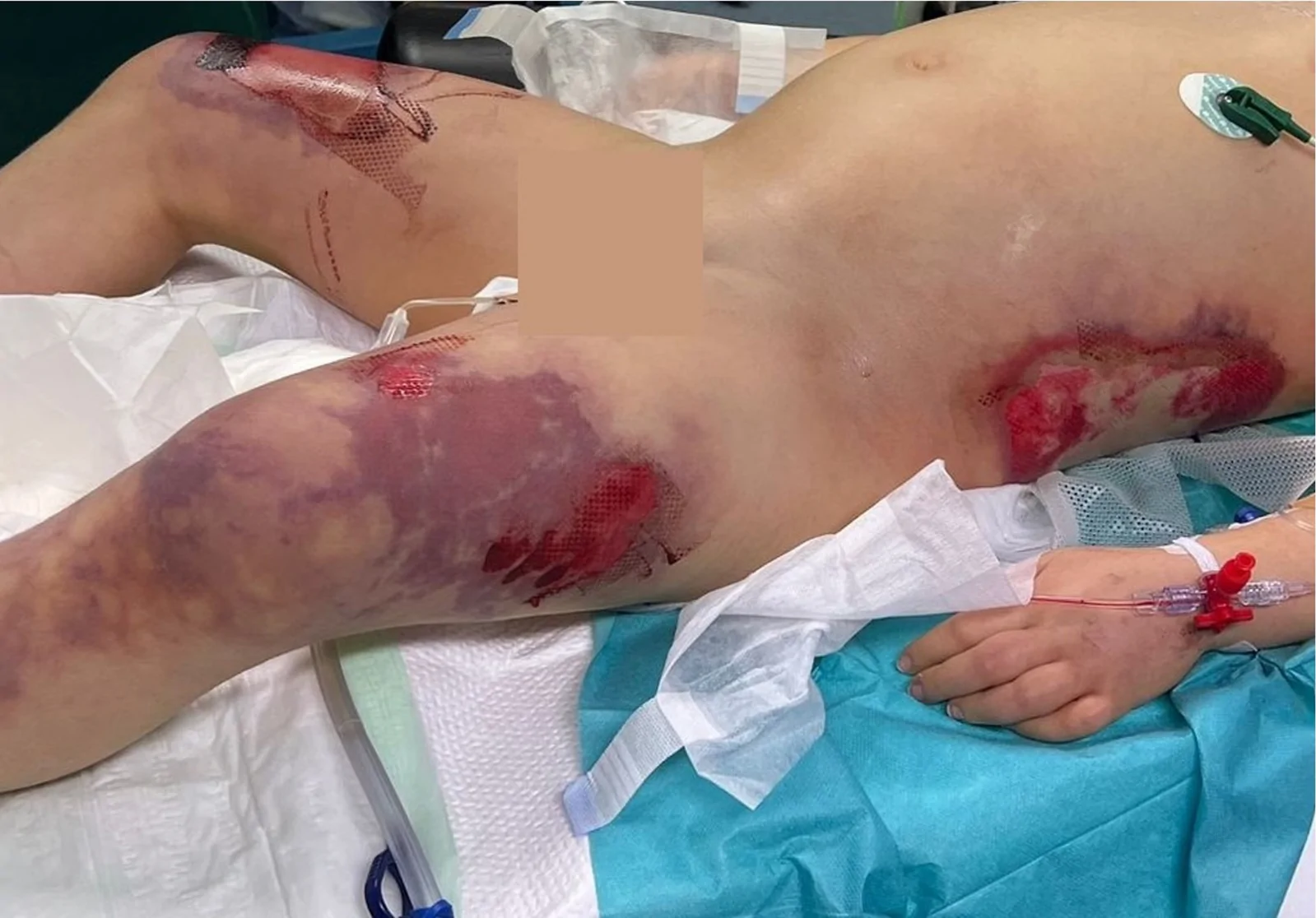
Cutaneous manifestations of Clostridium septicum infection upon arrival at our institution. Hemorrhagic, violaceous skin lesions with central necrosis on the left flank and thigh. The findings were consistent with clostridial myonecrosis. The skin changes had developed within hours and were accompanied by crepitus and systemic deterioration

Upon arrival at our pediatric intensive care unit, the patient presented with critical sepsis-associated multiorgan dysfunction and respiratory failure. Physical examination revealed a markedly distended and silent abdomen, generalized edema, and extensive skin lesions on both thighs and the left flank with a foul odor. The lesions appeared violaceous to hemorrhagic and were surrounded by zones of necrotic tissue (Fig. 2). All toes on the left foot were cyanotic and nonviable (Fig. 2). The patient presented with complete paraplegia.

**Fig. 3:**
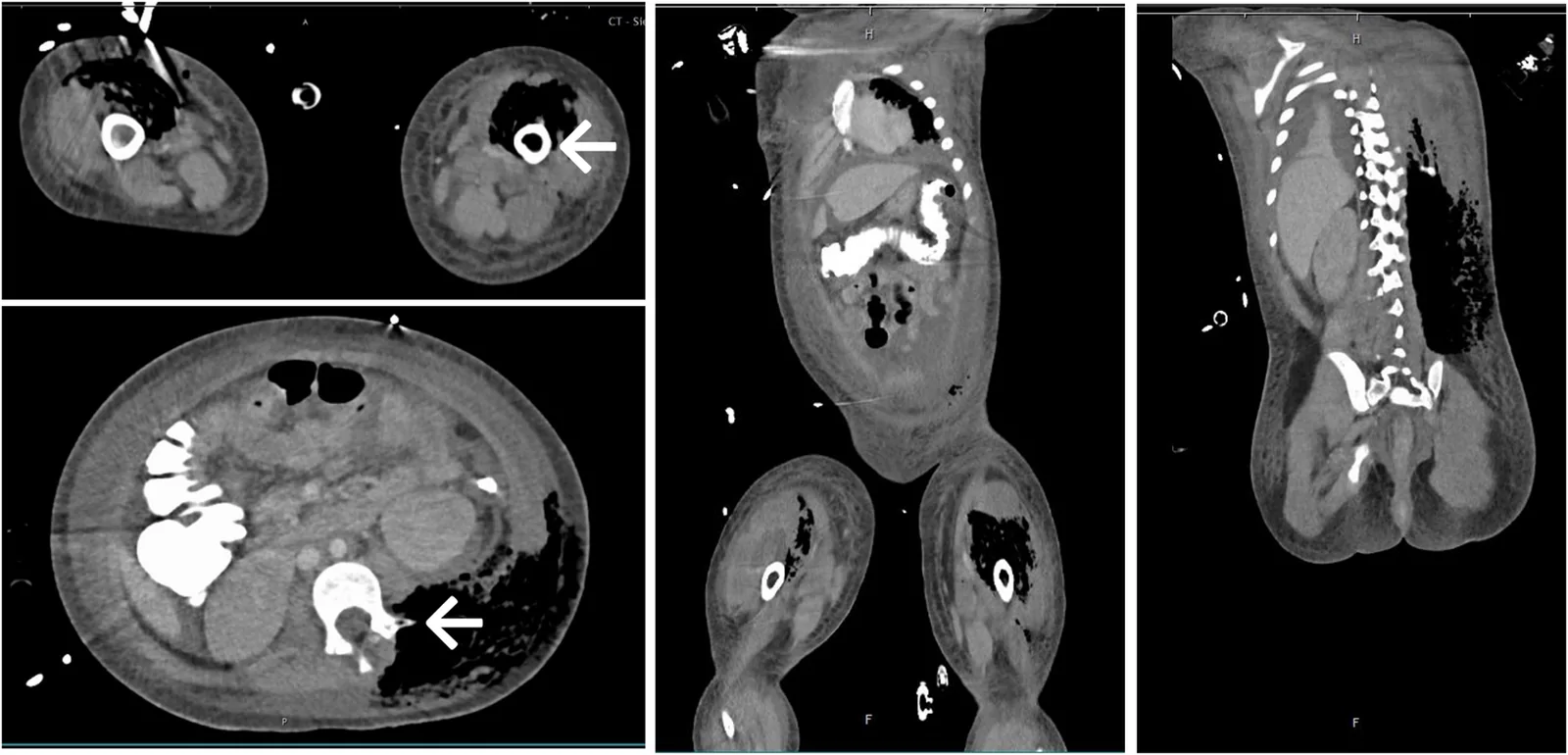
Contrast-enhanced CT scan upon admission to our institution. Extensive subcutaneous and intramuscular gas is visible in the left thoracic wall, posterior trunk, and bilateral thighs. Intraosseous gas inclusions are present in the femur and vertebral bodies (arrows). Additionally, marked thickening of the colonic wall and ascites indicate intraabdominal extension. These findings are consistent with clostridial myonecrosis with hematogenous dissemination and multi-compartimental involvement

A contrast-enhanced CT scan of the thorax and abdomen revealed extensive soft tissue emphysema (Fig. 3).

Based on the clinical presentation of a gas-forming soft tissue infection, the findings of the CT scan, and the microbiological detection of *C. septicum*, we diagnosed clostridial myonecrosis (gas gangrene) and established the indication for immediate radical surgical debridement as the only causal treatment option.

**Fig. 4:**
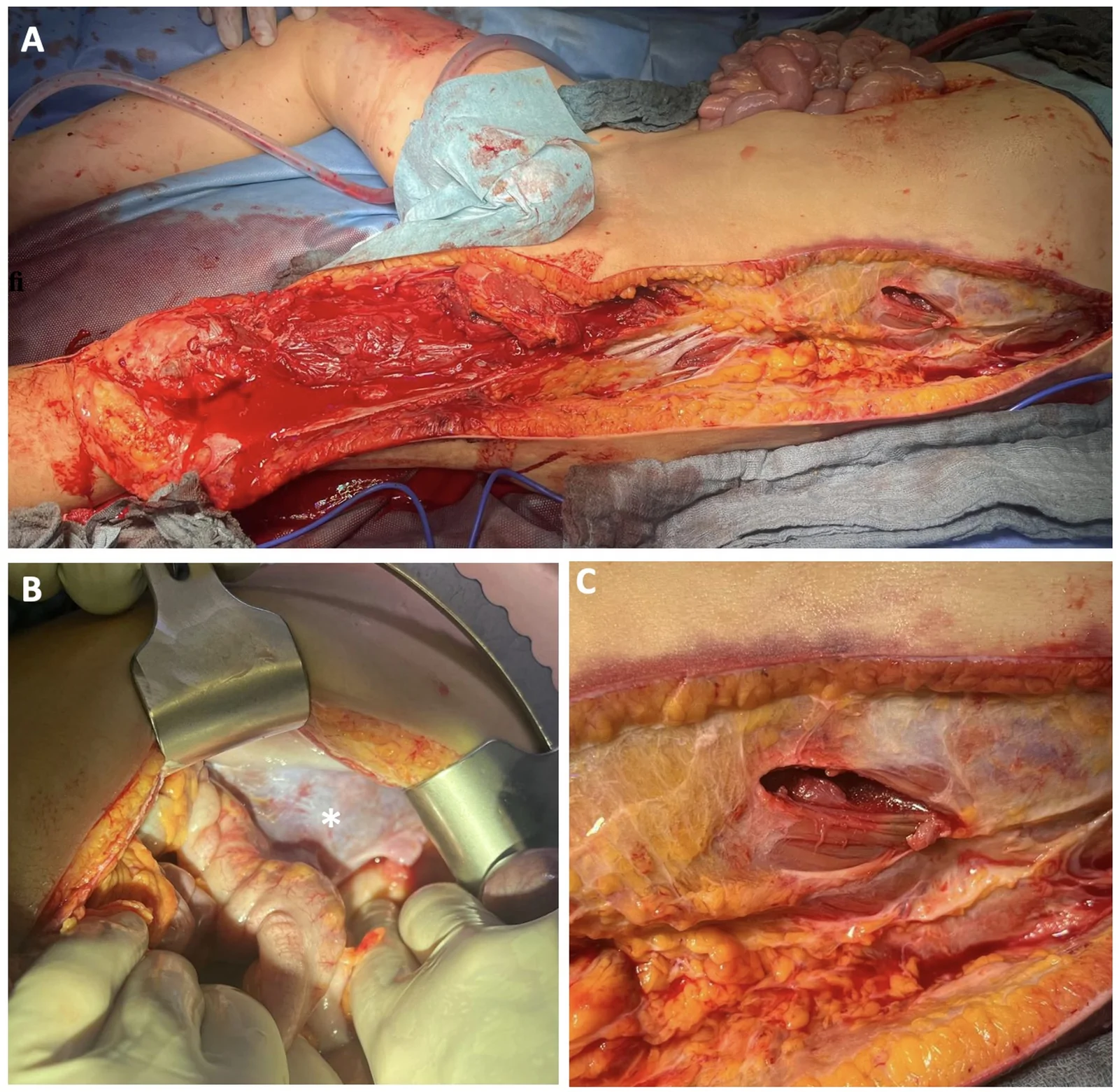
Intraoperative findings following midline laparotomy and debridement of the left lower extremity. **A** Extensive muscular necrosis of the left thigh with loss of tissue and minimal bleeding, consistent with clostridial myonecrosis. **B** Completely avital abdominal wall requiring minimal hemostasis; retroperitoneal involvement visible gas formation (*). **C** Macroscopic continuity of necrotic tissue from the abdomen to the thoracic wall and left thigh

The patient underwent simultaneous midline laparotomy and extensive debridement of the left lower extremity. Intraoperatively, the musculature of the left thigh appeared non-viable and exhibited minimal bleeding, with hemorrhage only from major vessels. Debridement was carried down to the femur (Fig. 4). The posterior compartment was also necrotic, indicating that fascial boundaries had not limited the spread of infection.

The laparotomy initially released copious, foul-smelling ascites, avital abdominal wall, devitalized intestines without overt ulceration or perforation. Retroperitoneal gas was visible on imaging and consistent with intraoperative findings (Fig. 4). The adrenal glands presented necrotic.

The necrotic areas in the abdomen and thigh were found to be continuous through the thoracic wall musculature (Fig. 4). Dissection from the abdomen to the iliopsoas muscle revealed complete necrosis of the psoas muscle.

Due to the extensive nature and inoperability of the infection, an interdisciplinary decision was made to shift the therapeutic goal to palliative care. The patient passed away shortly thereafter in the presence of her family.

## Discussion

Clostridial myonecrosis in children remains an exceptionally rare but devastating condition, typically associated with immunosuppression, gastrointestinal compromise, or hematologic malignancy. Its fulminant course, often characterized by subtle initial symptoms and rapid systemic deterioration, poses considerable diagnostic and therapeutic challenges in the pediatric population. Despite sporadic case reports over several decades, systematic data on presentation patterns, therapeutic timelines, and prognostic factors remain limited. Particularly pediatric infections with *C. septicum* demand special attention due to their high mortality and potential to progress without preceding trauma or evident entry point. Gastrointestinal involvement is increasingly reported and, particularly in the presence of ischemia or mucosal injury, may represent a potential site of origin for C. septicum infection. In this context, our study aims not only to synthesize all pediatric *C. septicum* cases reported since 2006 as an update to the previously published work by Smith-Slatas et al. (2006), but also to contribute clinical insights by presenting a fatal case in a previously healthy child with an unusual initial diagnosis of ileocolic intussusception. This combination—intussusception as a potential precursor or consequence of clostridial sepsis—has, to our knowledge, not been previously documented, and raises important questions about pathogenesis and early diagnostic thresholds.

While the landmark review by Smith-Slatas et al. provided the first structured overview of pediatric *C. septicum* infections, their analysis concluded in 2006 and was primarily descriptive, with limited focus on therapeutic timing and decision-making. Our study expands upon this by systematically compiling and analyzing all published cases since that time, thereby capturing developments in intensive care, surgical practice, and diagnostic imaging. Like earlier datasets, precise temporal correlations between symptom onset, antibiotic initiation, and surgical intervention could not be systematically analyzed, as the timing information provided in most reports was too vague (e.g., ‘within 24 hours’). This underscores the need for more detailed and standardized documentation of treatment timelines to better define the therapeutic window for clostridial myonecrosis in children.

When comparing our data set with the comprehensive review by Smith-Slatas et al., several notable shifts become apparent. Given the small sample size and the presence of several potential sources of bias, the following findings should be interpreted with caution.

Most notably, the proportion of cases associated with STEC-HUS has increased substantially and now accounts for half of all reported cases, which may reflect changes in epidemiology or reporting patterns over the past two decades. In parallel, the frequency of surgical intervention has risen considerably, and this correlates with a marked reduction in case fatality. These trends may reflect heightened awareness, earlier recognition of clostridial infections, and more aggressive surgical management. Additionally, the lower proportion of gastrointestinal involvement in our cohort could indicate broader detection across varying clinical presentations or shifts in underlying risk constellations. Together, these observations suggest that both, diagnostic vigilance and timely surgical response, have contributed to improved outcomes in recent pediatric cases of *C. septicum* infection, which is furthermore confirmed with the significant association of outcome and surgical treatment in the combined analysis of all published pediatric cases since 2006.

However, the observed increase in surgical intervention and its association with improved survival should be interpreted cautiously. Although patients who underwent surgical debridement had a higher observed survival rate, this finding does not establish a causal survival benefit. Survivorship bias may have contributed, as patients who died early may not have survived long enough to undergo surgery; thus, the association may partly reflect clinical stability and the opportunity to receive surgical treatment rather than the independent effect of debridement itself.

Moreover, gastrointestinal involvement, although common, was significantly associated with mortality, reinforcing the hypothesis that mucosal barrier disruption may facilitate systemic dissemination and complicate clinical management.

Among the 18 recent cases, a female predominance was observed (female ratio, 11:7); however, the limited sample size precludes firm conclusions, and this imbalance may represent a chance finding, reflect reporting bias, or indicate an underlying biological difference that cannot be established from the available data.

The current case describes a fatal C. septicum infection in a previously healthy child presenting with diarrhea, abdominal pain, and ileocolic intussusception.

The sequence of intussusception preceding the soft tissue infection is of particular interest. A single pediatric case of C. septicum infection associated with intussusception was reported in 1969; to our knowledge, the present case is the first describing recurrent re-intussusception [27]. Furthermore, intussusception is very atypical at the patient’s age.

It remains speculative whether the patient initially developed intussusception leading to localized bowel ischemia and mucosal injury with subsequent transmural bacterial translocation and CM, or whether an early, initially undetected intestinal C. septicum infection caused submucosal necrosis and edema that secondarily precipitated the invagination. Alternative pathophysiological mechanisms, including STEC-HUS as an underlying trigger, also cannot be excluded. Given the reported association between C. septicum infection and HUS, diarrhea-associated HUS complicated by C. septicum sepsis represents a possible, although unconfirmed, underlying pathophysiology.

The gastrointestinal tract remains a known origin of clostridial myonecrosis, typically in the setting of mucosal injury, ischemia, or bowel wall compromise—such as in malignancy, neutropenia, or obstruction [15, 28].

By the time our patient was transferred to our institution, more than 48 h had passed since symptom onset without targeted antimicrobial therapy or surgical intervention. To date, no published case has reported survival following such a delay in the treatment of clostridial myonecrosis [2–16].

Several important methodological limitations must be considered when interpreting our findings. First, the overall number of pediatric C. septicum cases remains extremely small, inherently limiting the robustness and interpretability of statistical analyses. Statistical associations derived from such rare-event datasets should be regarded primarily as exploratory observations rather than definitive predictors of outcome. Reported p-values may therefore overestimate the strength of associations. This systematic review was not prospectively registered, which should be considered a methodological limitation.

In addition, comparisons with the historical cohort published by Smith-Slatas et al. should be interpreted cautiously, as differences in inclusion criteria, reporting standards, case ascertainment, and therapeutic approaches over time may substantially affect comparability between datasets. Consequently, observed differences between cohorts cannot be interpreted as definitive epidemiological trends. Consequently, observed differences between cohorts cannot be interpreted as definitive epidemiological trends. Moreover, the pooled analysis of historical and recent cases should be interpreted with particular caution, as changes over time in diagnostic criteria, intensive care, imaging availability and quality, surgical practice, and publication patterns may have introduced substantial heterogeneity and may limit the validity of pooled estimates.

Furthermore, all included publications consisted of case reports or small case series, introducing several inherent sources of bias, including publication bias, selective reporting, heterogeneous diagnostic criteria, inconsistent reporting of treatment timelines, and variability in surgical and intensive care management. The inclusion of our previously unpublished institutional case in the pooled analysis, although it fulfilled all predefined eligibility criteria, may represent a potential source of bias. Given the descriptive nature of the available evidence, a formal quality scoring system was not applied. Nevertheless, reports were qualitatively assessed regarding diagnostic confirmation, completeness of clinical data, treatment description, and outcome reporting.

Important clinical variables were frequently incompletely documented, particularly regarding the exact timing of symptom onset, antimicrobial therapy, and surgical intervention. These limitations significantly restrict the ability to draw causal conclusions and underscore the descriptive nature of the present analysis. Nevertheless, given the exceptional rarity and high mortality of pediatric clostridial myonecrosis, systematic synthesis of available cases remains clinically valuable to identify potential presentation patterns, raise diagnostic awareness, and generate hypotheses for future investigation.

## Conclusion

In conclusion, clostridial myonecrosis should be suspected in any patient presenting with rapidly progressive soft tissue swelling, severe pain disproportionate to physical findings, skin discoloration, palpable crepitus, foul-smelling discharge, and early systemic signs such as fever and tachycardia. After the identification of gastrointestinal symptoms in early diagnosis of a prior review, we observed a significant association of the exclusion of gastrointestinal involvement and early surgical treatment with a positive outcome of the pediatric population in a combined analysis. A trend toward improved survival among surgically treated patients was observed. We suspect that earlier surgical intervention in patients with C. septicum infections is in general associated with a better chance of survival, but we were unable to confirm this due to insufficient data on the timing of surgery. Additionally, in cases of severe HUS, the association with *C. septicum* infections should be recognized and patients should be regularly examined regarding early clinical signs.

Our literature review finally emphasizes the devastating pace of C. septicum infection as well as the extremely narrow therapeutic window and highlights new possible early signs of gastrointestinal-associated risk factors.

## Supplementary Information

Supplementary Material 1.

Supplementary Material 2.

## Data Availability

All data generated or analysed during this study are included in this published article [and its supplementary information files].

## Abbreviations

AB: Antibiotics
AKI: Acute kidney injury
CM: Clostridial myonecrosis
CT: Computed tomography
GI: Gastrointestinal
NEC: Necrotizing enterocolitis
STEC-HUS: Hemolytic uremic syndrome associated with Shiga toxin-producing Escherichia coli
