# Supplementary material for: Pediatric Clostridium septicum infections: a systematic review and case-based perspective
Source: BMC Pediatr. 2026 Aug 22;26:802. doi: 10.1186/s12887-026-07544-5 (PMC13520299; doi:10.1186/s12887-026-07544-5)
Supplement: Supplementary file 2 [file 12887_2026_7544_MOESM2_ESM.docx]

**Identification of studies via other methods**

**Identification of studies via databases and registers**

Records identified from:

Websites:
- ClinicalTrials.gov (n = 0)

Organisations (n = 0)

Citation searching (n = 0)

- Reference screening of all included studies did not identify additional eligible publications.

Records removed *before screening*:

Duplicate records removed; Records removed for other reasons (n = 163)

Records marked as ineligible by automation tools (n = 0)

Records identified from*:

Databases (n = 206):

- Pubmed (n=103)
- Scopus (n = 71)
- Web of Science (n = 32)
- Cochrane library (n = 0)

Registers (n = 0)

**Identification**

Records excluded**

(n = 28)

Records screened

(n = 43)

Reports not retrieved

(n = 0)

Reports sought for retrieval

(n = 0)

Reports sought for retrieval

(n = 15)

Reports not retrieved

(n = 0)

**Screening**

Reports assessed for eligibility

(n = 0)

Reports assessed for eligibility

(n = 15)

Reports excluded (n= 0)

Due to the limited number of publications on the subject, all reports that met the inclusion criteria were included in the analysis.

Studies included in review

(n = 15)

Reports of included studies

(n = 17)

**Included**

*Consider, if feasible to do so, reporting the number of records identified from each database or register searched (rather than the total number across all databases/registers).

**If automation tools were used, indicate how many records were excluded by a human and how many were excluded by automation tools.

Source: Page MJ, et al. BMJ 2021;372:n71. doi: 10.1136/bmj.n71.

This work is licensed under CC BY 4.0. To view a copy of this license, visit <https://creativecommons.org/licenses/by/4.0/>
